# Unraveling the Multilayered Regulatory Networks of miRNAs and PhasiRNAs in *Ginkgo biloba*

**DOI:** 10.3390/plants14111650

**Published:** 2025-05-29

**Authors:** Qixuan Wei, Ang Xu, Anqi Zhao, Lisha Shi, Qi Wang, Xiaoming Yang, Meiling Ming, Liangjiao Xue, Fuliang Cao, Fangfang Fu

**Affiliations:** 1 State Key Laboratory of Tree Genetics and Breeding, Co-Innovation Center for Sustainable Forestry in Southern China, Nanjing Forestry University, Nanjing 210037, China; weiqixuan@njfu.edu.cn (Q.W.); angxa@nifu.edu.cn (A.X.); anqi@njfu.edu.cn (A.Z.); sls@njfu.edu.cn (L.S.); qwang@njfu.edu.cn (Q.W.); xmyang@njfu.edu.cn (X.Y.); mingmeiling@njfu.edu.cn (M.M.); fuliangcaonjfu@163.com (F.C.); 2 The Jiangsu Province Platform for Construction and Utilization of Agricultural Germplasm, Nanjing 210037, China

**Keywords:** *Ginkgo biloba*, miRNA-PHAS modules, reproductive development, flavonoid biosynthesis, disease resistance, regulatory network

## Abstract

Small RNAs (sRNAs) are pivotal in regulating gene expression and are involved in a diverse array of biological processes. Among these, microRNAs (miRNAs) and phased small interfering RNAs (phasiRNAs) have been extensively investigated over the past decades. We conducted an in-depth analysis of deep sequencing data from the gymnosperm *Ginkgo biloba*, encompassing sRNA, transcriptome, and degradome libraries. Our analysis identified a total of 746 miRNAs and 654 phasiRNA precursor (PHAS) loci, with 526 (80%) of the PHAS loci predicted to be triggered by 515 miRNAs (69%). Several miRNA-PHAS modules, particularly the miR159/miR319-PHAS module, were found to potentially regulate reproductive development by targeting *GAMYB* genes and triggering phasiRNA biogenesis. The miR390-PHAS module appears to be involved in flavonoid biosynthesis by targeting key enzyme genes such as chalcone synthase (*CHS*) and anthocyanin synthase (*ANS*). Through target gene identification and coexpression analysis, we uncovered two distinct models of complex regulatory networks: growth-related factors like *ARF* and *GRF* seem to be regulated exclusively by miRNAs (Model 1), while certain disease resistance-related genes are predicted to be regulated by both miRNAs and phasiRNAs (Model 2), indicating diverse regulatory mechanisms across different biological processes. Overall, our study provides a comprehensive annotation of miRNA and PHAS loci in *G. biloba* and elucidates a post-transcriptional regulatory network, offering novel insights into sRNA research in gymnosperms.

## 1. Introduction

Small RNAs (sRNAs) are critical post-transcriptional regulators in plants, typically ranging from 20 to 24 nucleotides (nt) in length. They precisely modulate gene expression and play a pivotal role in key stages of plant development and responses to environmental cues [1,2]. Among sRNAs, microRNAs (miRNAs) and phased small interfering RNAs (phasiRNAs) are extensively studied due to their essential roles in plant growth and reproductive processes.

miRNAs are central to post-transcriptional gene silencing, targeting specific mRNAs to influence a wide range of developmental pathways. For example, miR156-*SPL*, miR172-*AP2*, miR159-*GAMYB*, and miR390-*ARF* are involved in the precise regulation of vegetative phase transitions, floral organ formation, and flower development [3,4,5,6]. Additionally, miRNAs play crucial roles in hormone signaling pathways, such as the involvement of miR393 in auxin and jasmonic acid signaling [7]. In *Larix leptolepis*, miR156 may be involved in ABA-induced regulation of embryogenesis [8]. On the other hand, phasiRNAs operate through a secondary siRNA synthesis mechanism triggered by miRNAs. They not only regulate plant development but also play vital roles in stress responses by participating in gene silencing and chromatin remodeling [9]. For instance, 24-nt phasiRNAs are essential for spikelet formation through the regulation of embryonic development and reproductive cell formation [10]. Therefore, the regulatory networks of miRNAs and phasiRNAs collaboratively govern the intricate mechanisms underlying plant development and responses to environmental stress [7,11].

Despite the increasing understanding of sRNA-mediated regulation, gymnosperms, including *Ginkgo biloba*, remain less explored compared to angiosperms. *G. biloba*, which has existed on Earth for over 200 million years, is often referred to as a “living fossil” and serves as a unique model for studying ancient plant lineages. *G. biloba* extract (GBE), particularly flavonoids and terpene trilactones (TTL), have garnered attention for their medicinal properties, especially in the treatment of cardiovascular diseases [12]. Beyond its medicinal value, *G. biloba* plays an essential role in ecological conservation, highlighting the importance of understanding its reproductive development and how regulatory mechanisms influence the synthesis of these bioactive compounds.

Previous research on the sRNA in *G. biloba* has primarily focused on the regulatory roles of miRNAs [13,14]. In the leaves of *G. biloba*, researchers have concentrated on the functions of miR160 and miR5261 in regulating hormone signaling pathways by targeting auxin response factors (*ARFs*) and brassinosteroid insensitive 1 (*BRI1*) [15]. Studies of *G. biloba* ovules, embryos, and cones have implicated miR156 in the regulation of morphophysiological dormancy, particularly in gibberellin (GA) biosynthesis and signaling [16]. miR482 and miR2950 were found to be highly expressed during the dormancy period of the cambium [17]. Additionally, studies have indicated that the long non-coding RNA (lncRNA)-miRNA gene network is involved in the synthesis of flavonoids [18,19]. However, the role of phasiRNAs in *G. biloba* remains poorly understood. PhasiRNAs, which are prevalent in other plant species, may hold the key to unraveling *G. biloba’s* post-transcriptional regulatory networks, particularly in reproductive tissues and secondary metabolite biosynthesis. Investigating these regulatory sRNAs presents an opportunity to uncover novel mechanisms that govern reproductive development and stress responses in *G. biloba*.

This study aims to establish a comprehensive sRNA-mediated regulatory network at the whole-genome level in *G. biloba*, utilizing transcriptome, small RNA, and degradome-supported bioinformatics analyses. This network provides a reference framework for evaluating sRNA-mediated regulation in growth, pathogenesis, and various environmental conditions. Ultimately, it elucidates the role of sRNAs in genome-wide gene expression circuits, the post-transcriptional regulatory mechanisms governing flower development, and the involvement of phasiRNAs in flavonoid biosynthesis in *G. biloba*. We conducted an in-depth analysis of miRNAs and phasiRNAs, along with their target genes, shedding light on their broader functions across plant species and physiological processes. These findings offer new insights into the miRNA regulatory networks in gymnosperms.

## 2. Materials and Methods

### 2.1. Plant Materials and Data Sources

For small RNA sequencing, *Ginkgo biloba* leaves were collected from 2-year-old clonally propagated (grafted) trees grown at three different experimental sites, with three replicates at each site [20]. The filtered data have been deposited in the NCBI database (PRJNA903548). Additionally, 25 small RNA datasets of *G. biloba* were downloaded from the NCBI Sequence Read Archive (SRA), which include eight samples of flowers (female flower buds (FB), ovulate strobilus (OS), male flower buds (MB), and microstrobilus (MS)) [13], two samples of ovules [14], nine samples of embryos (collected in September, November, and February, respectively) [21], and six samples of cambia at different ages (15, 20, 193, 211, 538, and 553 years) [22] (Table S1).

A total of 34 sRNA-seq and 42 RNA-seq datasets from studies with different experimental goals were used, raising the potential for batch effects caused by biological (e.g., tree variety, growth conditions) and technical (e.g., RNA extraction, library preparation, sequencing platform) variability. To mitigate these effects and ensure data comparability, we normalized small RNA reads to reads per 10 million (RP10M), applied log2 transformation to reduce skewness, and standardized expression values across samples to focus on relative differences within datasets.

### 2.2. sRNA Library Construction and Alignment Analysis

Total RNA was extracted from nine freeze-dried leaf samples using the Trizol reagent kit (Invitrogen, Carlsbad, CA, USA), following the manufacturer’s protocol. Sequencing was subsequently performed using Illumina Hiseq2000/2500 platform (San Diego, CA, USA) with 50 bp single-end model.. The sequencing data were initially preprocessed by removing adapter sequences, short reads, and low-quality reads, as well as discarding sequencing fragments with read lengths shorter than 18 nt. Redundant sequences were then merged. Next, non-coding RNAs (rRNAs, snoRNAs, and tRNAs) from the chloroplast and mitochondrial genomes were filtered out by mapping against Rfam V13.0 (https://rfam.org/) [23] and the Plant Organelles Database (http://podb.nibb.ac.jp/Organellome/podb3/search.php, accessed on 27 March 2024) [24]. This process yielded high-quality sequencing fragments, referred to as clean data.

Using Bowtie [25] invoked by the sRNAminer software (v.1.1.2) [26], the clean data from small RNA sequencing were aligned to the genome of *G. biloba* previously published by our research group [27]. Due to the large size of the genome, we modified the command (bowtie-build {input_genome} {input_genome}—large-index) to prevent indexing errors. This adjustment enabled us to obtain the positional information and annotations of the mapped reads on the reference genome. Up to two mismatches were permitted in the alignment for subsequent analysis.

### 2.3. miRNA and PHAS Loci Identification

In the sRNAminer methodology [26], the identification of miRNA and PHAS loci follows specific criteria: miRNA loci must exhibit a spacing of 5 to 300 nucleotides between the mature miRNA and the miRNA star, with the mature miRNA length ranging from 20 to 22 nucleotides. Additionally, sRNAs with more than 20 genomic hits are filtered out to minimize interference from repetitive sequences. The combined abundance of the miRNA and miRNA star must constitute at least 75% of the total reads from the MIR locus. Known miRNAs are named based on homology, while newly identified miRNAs are prefixed with “miRN”. The miRNA with RP10M < 10 in certain samples was labeled as “probably” in Table S2.

For PHAS loci, the following conditions must be satisfied: (1) the predominant siRNA length is either 21 nt or 24 nt; (2) the abundance of phasiRNAs exceeds 30%; (3) the locus length is greater than 100 bp; and (4) the *p*-value is less than 0.001, with a phasing score greater than 10. Trigger miRNAs are identified by aligning them to the PHAS loci and their flanking sequences (totaling 400 bp), with an alignment penalty score of ≤5.

### 2.4. Screening of Differential Expressed miRNAs and PHAS

The expression levels of each sRNA were quantified as Reads Per 10 Million (RP10M). For the heatmap, a Z-score normalization was performed on the normalized RP10M for each sRNA. Z-scores were computed on an sRNA-by-sRNA (row-by-row) basis by subtracting the mean and then dividing by the standard deviation [11]. Only the miRNAs or phasiRNAs with RP10M >10 in certain samples were used for further analysis. Differential expression analysis was performed by edgeR (v3.34.0) [28] to compare reproductive tissues (ovule, embryo, FB, OS, MB, MS) with vegetative tissues (leaf, cambium). Differentially expressed miRNAs were identified based on false discovery rate (FDR) and fold change (FC) criteria, with thresholds set at |log2(FC)| ≥2 and FDR ≤0.05 for miRNAs, and |log2(FC)| ≥1 and FDR ≤0.05 for PHAS loci.

### 2.5. Identification of Target Genes for miRNA and phasiRNA

We used the GSTAr software (https://github.com/MikeAxtell/GSTAr, accessed on 27 March 2024, v1.0) to predict potential binding sites between RNA molecules, applying the following filtering thresholds: MFE ratio > 0.65 and Allen Score < 7. Subsequently, we employed TargetFinder (v1.0.0, −v 0) for target gene prediction. The intersection of predictions from GSTAr and TargetFinder, where miRNA-mRNA pairs corresponded with identical target gene sequences, was considered reliable for further analysis. For functional annotation, we used BLAST software (v2.2.26, −b 100 -v 100 -e 1e-5 -m 7 -a 2) to compare target genes against the NCBI non-redundant (nr) database, Swiss-Prot [29], Gene Ontology (GO) [30], and the Kyoto Encyclopedia of Genes and Genomes (KEGG) [31]. GO and KEGG enrichment analyses of target genes were conducted using the R package clusterProfiler (version 3.18.1) [32], with FDR ≤0.05. Degradome data were used to validate miRNA and phasiRNA cleavage sites. CleaveLand 4.0 was applied to identify target genes cleaved by miRNA and phasiRNA. Target genes were classified into five categories (0–4), with transcripts in categories 0–3 within a single library and an alignment penalty score ≤6 considered reliable cleavage sites.

The Fuzzy C-Means Clustering (FCM) algorithm from the R package MFUZZ [33] was employed to cluster genes and miRNAs.

### 2.6. Identification of Regulatory Networks

The PHAS triggers (miRNA-phasiRNA) identified using sRNAminer [26] were integrated with the intersected results of GSTAr and TargetFinder for reliable target gene prediction (miRNA-mRNA and phasiRNA-mRNA). This process ultimately established the miRNA-phasiRNA-mRNA interaction relationships. Subsequently, edgeR (v3.34.0) [28] was used to calculate the fold changes (FoldChange) for all miRNAs, mRNAs, and PHAS loci by comparing reproductive tissues (ovule, embryo, FB, OS, MB, MS) with vegetative tissues (leaf, cambium) under the threshold of FDR ≤0.05 (RP10M >10 in certain samples). The fold changes of all miRNAs, PHAS loci, and mRNAs were then mapped to the miRNA-phasiRNA-mRNA interaction pairs to serve as the basis for subsequent filtering.

We defined the criteria for Model 1, in which regulation is solely miRNA-mediated without phasiRNA involvement, as follows: (1) log2FC (miRNA) × log2FC (PHAS loci) < 0 or |log2FC (PHAS loci)| < 1, ensuring that phasiRNAs are not triggered by miRNAs or phasiRNAs are not differentially expressed. (2) log2FC (miRNA) × log2FC (mRNA) < 0, with |log2FC (miRNA)| > 1 and |log2FC (mRNA)| > 1, confirming miRNA-mediated downregulating of target mRNA (via cleavage or translational repression).

For Model 2, in which both miRNA and phasiRNA collaboratively mediate regulation, the criteria were defined as: (1) log2FC (miRNA) × log2FC (PHAS loci) > 0 and |log2FC (PHAS loci)| > 1, ensuring that phasiRNAs are either triggered by miRNAs or are differentially expressed. (2) log2FC (miRNA) × log2FC (mRNA) < 0, with |log2FC (miRNA)| > 1 and |log2FC (mRNA)| > 1, confirming miRNA-mediated downregulating of target mRNA (via cleavage or translational repression). To avoid false negatives caused by expression differences in reproductive or vegetative tissues, we manually inspected the data for typical conserved miRNA families to confirm data reliability.

The filtered miRNA-mRNA interactions and miRNA-phasiRNA interactions with triggering relationships combined with high expression correlation (r > 0.7) were visualized using Cytoscape.

### 2.7. Degradome Sequencing

The female and male flower buds collected at the end of March, 2022 were mixed and used for the degradome sequencing. Total RNA was isolated and purified using TRIzol reagent (Invitrogen, Carlsbad, CA, USA) according to the manufacturer’s instructions. The quantity and purity of each RNA sample were quantified using a NanoDrop ND-1000 (NanoDrop, Wilmington, DE, USA). RNA integrity was assessed by an Agilent 2100 Bioanalyzer (Santa Clara, CA, USA), with a RIN number greater than 7.0. Poly(A) RNA is purified from 20 μg of total plant RNA using poly-T oligo-attached magnetic beads through two rounds of purification. Due to the presence of a 5′-monophosphate on the 3′ cleavage product of the mRNA, 5′ adapters were ligated to the 5′ end of this product using RNA ligase. The subsequent step involved reverse transcription to synthesize the first strand of cDNA using a 3′-adapter random primer, followed by size selection with AMPure XP beads (Brea, CA, USA). The cDNA was then amplified via PCR under the following conditions: initial denaturation at 95 °C for 3 min; 15 cycles of denaturation at 98 °C for 15 s, annealing at 60 °C for 15 s, and extension at 72 °C for 30 s; concluding with a final extension at 72 °C for 5 min. The average insert size of the final cDNA library was 200–400 bp. Finally, we performed 50 bp single-end sequencing on an Illumina HiSeq 2500 (LC Bio, Hangzhou, China) following the vendor’s recommended protocol. The degradome data accession ID is PRJCA038234 (National Genomics Data Center, Beijing, China). The target genes validated by degradome analysis for miRNA, 21-nt phasiRNA, and 24-nt phasiRNA are provided in Tables S9, S10 and S11, respectively.

## 3. Results

### 3.1. Identification of miRNA and PHAS Loci

**Figure 1:**
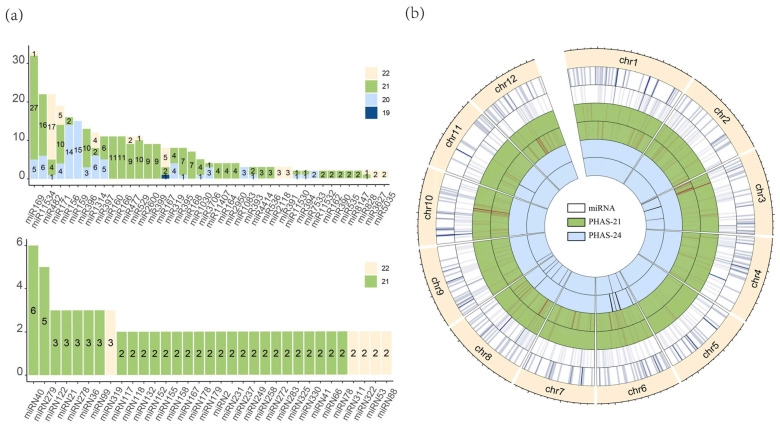
Characterization of miRNAs and PHAS loci in *G. biloba*. (**a**) The number of members in each known miRNA (upper panel) and novel miRNA family (lower panel) is presented. The color bar on the right indicates the different lengths of miRNAs. (**b**) A heatmap illustrates the chromosomal distribution of all miRNA and PHAS loci, each with miRNA-phasiRNA trigger (outer) and non-trigger (inner) relationship.

To systematically investigate post-transcriptional regulation during the development of *Ginkgo biloba*, we obtained and analyzed small RNA sequencing (sRNA-seq) data from 19 samples of four reproductive tissues (ovules, embryos, male flowers, and female flowers) and 15 samples of two vegetative tissues (leaves and cambia) (Table S1). A total of 623.03 million raw sRNA reads and 55.5 million clean reads were generated. Through sRNAminer analysis, we identified 746 miRNAs from the *G. biloba* genome, which included 342 known miRNAs belonging to 66 miRNA families and 404 novel miRNAs derived from 358 novel MIR loci (Table S2). The most abundant miRNA families were miR169 (32 members), miR482 (25 members), and miR11534 (24 members). The miR428 family predominantly consisted of 22-nt members, while the miR156 and miR159 families were primarily composed of 20-nt members, with most other families comprising 21-nt miRNAs (Figure 1a).

We identified a total of 620 PHAS loci generating 21-nt phasiRNAs and 34 PHAS loci producing 24-nt phasiRNAs, all with a maximum phasing score exceeding 10 (Table S3). Of these, 526 PHAS loci were found to be triggered by miRNAs (Table S4). A total of 515 miRNAs, belonging to families such as miR1314, miR11534, miR159, and miR482, were identified as triggers (Table S4). The remaining PHAS loci, which lack consistent miRNA triggers, may be processed through alternative, unknown mechanisms. Our analysis revealed that the majority of miRNAs and PHAS loci exhibit targeting relationships, with certain miRNA families consistently triggering specific PHAS loci. For example, all members of the miR171 family triggered PHAS21-68, the miR167 family triggered PHAS21-27, and the miR319 family triggered PHAS21-555 (Table S4). The miR482 family was particularly prolific, with nearly every member capable of triggering multiple PHAS loci. Notably, the 21-nt PHAS loci were predominantly located on chromosomes 3, 10, and 12 (Figure 1b). Both miRNA-triggered and non-miRNA-triggered PHAS loci were enriched at the same genomic locations, suggesting that PHAS loci triggers are influenced by the genomic positions of the loci as well as the involvement of miRNAs.

### 3.2. Expression Patterns of miRNA and PHAS Loci

**Figure 2:**
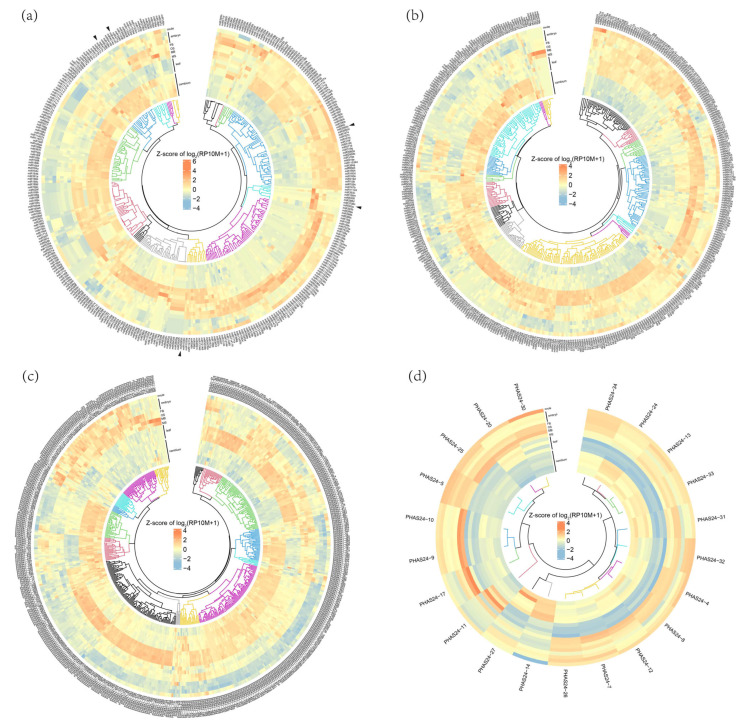
The expression profiles of all miRNAs and PHAS loci across various tissues. The expression levels of known miRNAs (**a**), novel miRNAs (**b**), 21-nt phasiRNAs (**c**), and 24-nt phasiRNAs (**d**) were normalized as Reads Per 10 Million (RP10M). Black arrows indicate the miRNAs specifically described in this paragraph. Z-scores were calculated for each sRNA and then plotted for the heatmap. The tissues include ovules, embryos at different developmental stages (collected in September, November, and February, respectively), female flower buds (FB), ovulate strobili (OS), male flower buds (MB), microstrobili (MS), leaves from different environments, and cambium from trees of different ages (15, 20, 193, 211, 538, and 553 years) (Table S1).

The expression patterns of all miRNAs and PHAS loci were analyzed across eight distinct tissues derived from 34 libraries (Table S1). The findings indicated that numerous miRNAs were highly expressed in both leaves and reproductive tissues, whereas cambium exhibited a distinctly different expression profile (Table S2). Notably, miRNAs that were highly expressed in the cambium, such as the entire miR477 and miR4414 families, were nearly absent in reproductive tissues, suggesting their potential role in xylem and phloem differentiation and development. Additionally, several miRNA families displayed consistent expression patterns: all members of the miR393 family were expressed in male buds (MB) and male flowers (MS), the entire miR2950 family was highly expressed in reproductive tissues, and all members of the miR2600 family showed high expression in leaves (Figure 2a,b, Figures S1 and S2). Regarding phasiRNAs, the analysis revealed that 21-nt phasiRNAs exhibited more tissue-specific expression, being exclusively present in leaves, cambia, or reproductive tissues (Figure 2c and Figure S3). In contrast, 24-nt phasiRNAs were all highly expressed in reproductive tissues but not in leaves, with some also showing expression in the cambium (Figure 2d and Figure S4, and Table S3).

### 3.3. Reproductive Tissues Preferentially Expressed miRNAs and PHAS Loci

**Figure 3:**
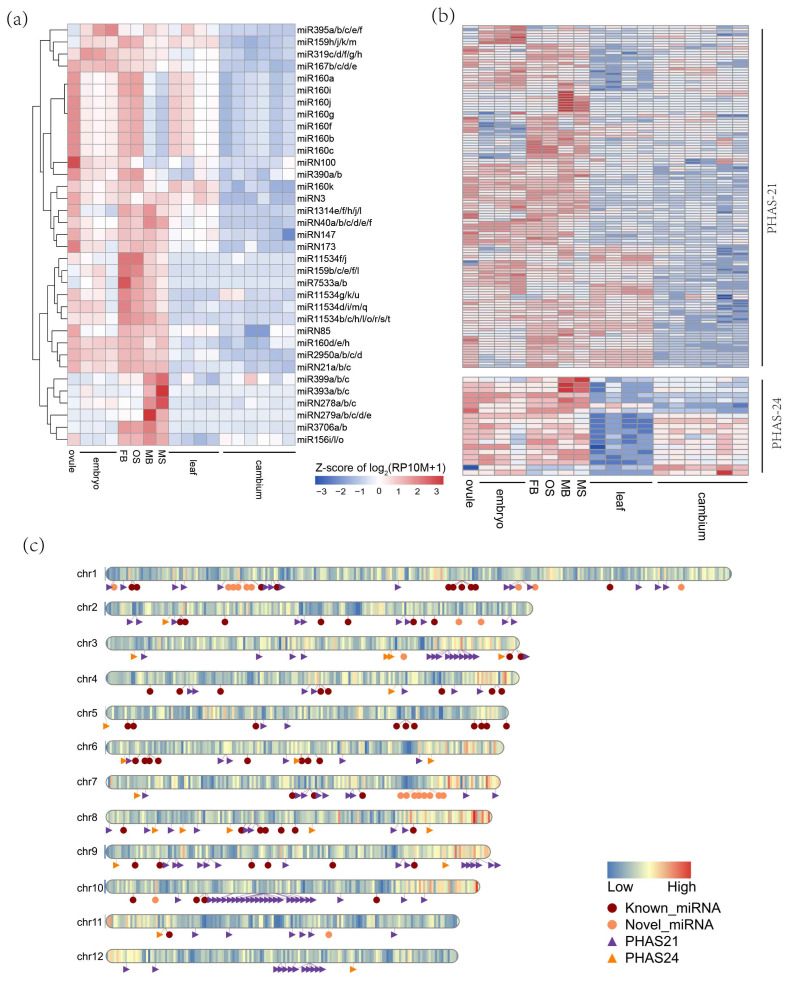
Reproductive tissue preferentially expressed miRNAs and PHAS loci. The differential expression between reproductive tissues (ovule, embryo, FB, OS, MS, MS) and vegetative tissues (leaf, cambium) were analyzed. The thresholds were set at |log2FC| > 2 for miRNAs and |log2FC| > 1 for phasiRNAs, and FDR < 0.05. (**a**) Expression profile of miRNAs (RP10M > 10 in certain samples). (**b**) Expression profile of 21-nt and 24-nt phasiRNAs. (**c**) The distribution of miRNAs and phasiRNAs along the chromosomes. Z-scores were calculated for each sRNA and then plotted for the heatmap.

Among all the miRNAs analyzed, 102 miRNAs (79 known and 23 novel) were found to be highly expressed in reproductive tissues compared to the vegetative tissues (fold change > 4, RP10M > 10, FDR < 0.05, across multiple libraries) (Figure 3a). Specifically, miR1314e/f/h/j/l, and miR3706a/b were highly expressed during the early stages of male flower development (MB). As the male flower matured (MS), miR393a/b/c and miR399a/b/c gradually accumulated, suggesting a potential role for these miRNAs in male flower development. Additionally, the entire miR7533 family, the miR2950 family, nearly all members of the miR11534 family (17 out of 24), and most of the miR159 family (9 out of 15) were highly expressed during the early stages of female flower development (FB), indicates a possible role for these miRNAs in the development of *G. biloba* female flowers. Moreover, miR395a/b/c/e/f/i were progressively activated as the embryo developed, suggesting their involvement in embryo development. Beyond the conserved miRNAs, eight novel miRNAs were also predominantly expressed in reproductive tissues (Figure 3a).

Based on the abundance of each PHAS locus, we identified 133 21-nt phasiRNAs and 21 24-nt phasiRNAs that were preferentially expressed in reproductive tissues (fold change > 2, RP10M > 10, FDR < 0.05, across multiple libraries) (Figure 3b), suggesting their potential regulatory roles in the development of *G. biloba* reproductive organs. Among those, 21-nt phasiRNAs were almost absent in the cambium and sparsely expressed in leaves, while 24-nt PHAS loci showed the opposite pattern, being nearly absent in leaves and expressed in the cambium (Figure 3b). This observation indicates a functional differentiation between 21-nt and 24-nt PHAS loci.

Next, we examined the chromosomal distribution patterns of the 97 miRNAs and 154 phasiRNAs that were preferentially expressed in reproductive tissues (Figure 3c). The 24-nt PHAS loci were found to be randomly scattered across the chromosomes, whereas 21-nt PHAS loci were predominantly concentrated on chromosomes 3, 10, and 12, with a notable enrichment observed on chromosome 10. Interestingly, aside from the sRNAs on chromosome 10, the majority of sRNAs were located in regions with high gene density. This suggests that chromosome 10 may be rich in repetitive sequences and transposable elements, which could necessitate the presence of PHAS loci in this region to produce phasiRNAs for transposon silencing.

**Figure 4:**
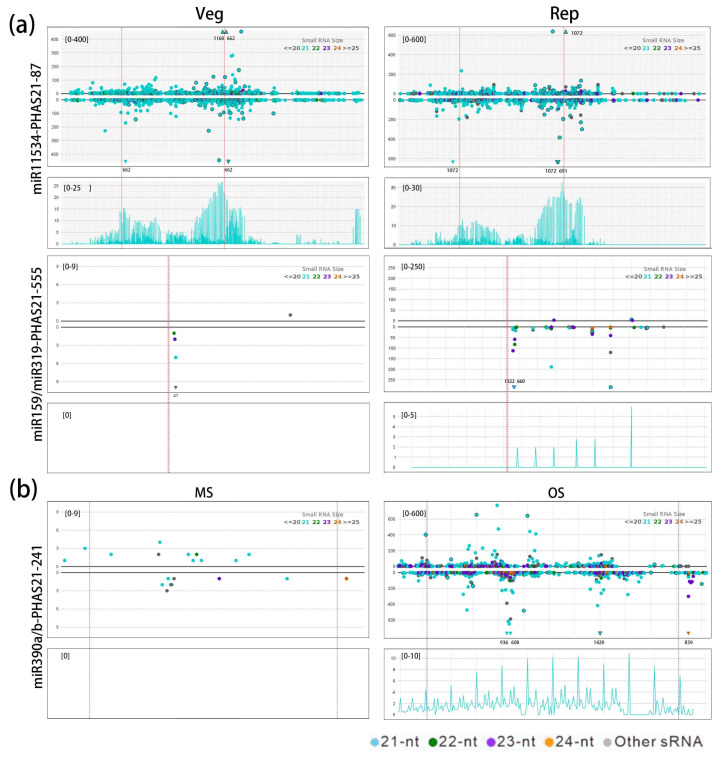
The triggering relationship of miRNA-phasiRNA pairs. (**a**) miR11534 triggers the PHAS21-87 in both vegetative (Veg) and reproductive (Rep) tissues, while miR159/319 triggers PHAS21-555 only in reproductive tissues. The upper section represents the abundance of sRNAs, and the lower section displays the phasing score. The y-axis scale indicates abundance, with differently colored dots representing sRNAs of varying lengths. The dashed lines mark the cleavage sites triggered by miRNAs, with red indicating the positive strand and blue indicating the negative strand. Triangular markers represent sRNAs with abundances that exceed the y-axis range. (**b**) miR390a/b triggers PHAS21-241 in both male (MS) and female (OS) flowers, with higher expression levels observed in female flowers.

Upon analyzing the triggers for these miRNAs and PHAS loci, we identified several miRNA-PHAS pairs that are preferentially expressed in reproductive tissues (Table S5). For example, the miR11534 family triggers phasiRNA production at PHAS21-87 and PHAS21-587, a pattern also observed with miR7533a/b. Nearly all members of the miR159 and miR319 families trigger phasiRNA production at the PHAS21-555 locus. Among the novel miRNAs, all members of the miRN40 family trigger phasiRNA production at the PHAS24-8 locus. Our analysis revealed that phasiRNA expression patterns are influenced by their triggering miRNAs and the expression of their targeted PHAS loci. For instance, PHAS21-87 and its triggering miRNAs, miR11534a/b/c/f/g/h/o/v, accumulate in both reproductive and vegetative tissues (Figure 4a). In contrast, PHAS21-555, co-triggered by the miR159 family (11 members) and miR319 family (7 members), is highly expressed exclusively in reproductive tissues (Figure 4a). Additionally, some PHAS loci exhibit differential expression between male and female flowers. For example, PHAS21-241, targeted by miR390ab, shows distinct expression patterns in male and female flowers (Figure 4b), suggesting that these PHAS loci may be involved in the development of male or female reproductive tissues.

### 3.4. The Target Genes of miRNAs and phasiRNAs in G. biloba

**Figure 5:**
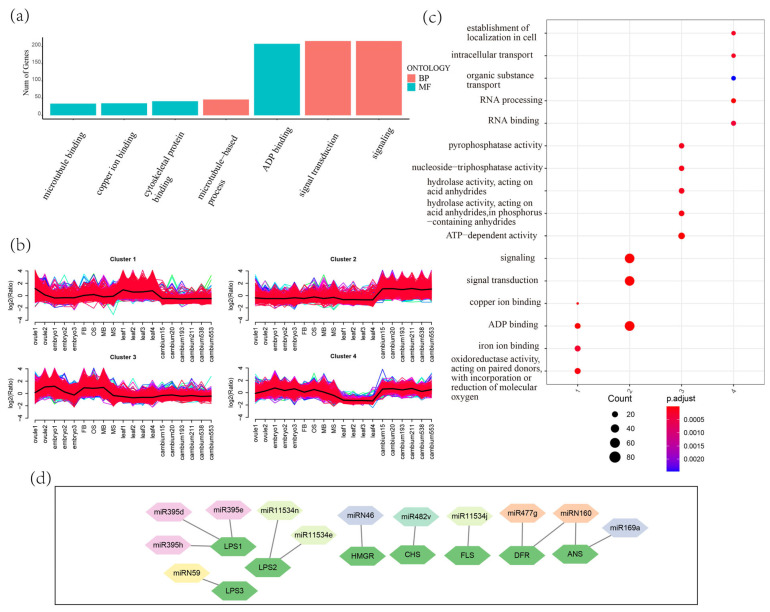
miRNA target genes in *G. biloba*. (**a**) Top Gene Ontology (GO) categories of all miRNA target genes. (**b**) Target genes were clustered into four distinct groups using the Fuzzy C-Means (FCM) algorithm from the R package MFUZZ, based on their expression patterns across different tissues. The x-axis represents the tissues, while the y-axis displays log2-transformed, normalized intensity ratios. (**c**) GO enrichment analysis of miRNA target genes in each cluster. (**d**) Flavonoids and terpene trilactones biosynthesis genes targeted by miRNAs in *G. biloba*.

We used GSTAr to predict RNA binding sites, followed by TargetFinder refinement, yielding 9934 predicted interactions. In total, 731 candidate miRNAs (340 known, 391 novel) were found to target 3610 annotated coding genes (Table S6). For phasiRNAs, the top 30 most abundant phasiRNAs from each PHAS locus were used for analysis, and 6924 target genes were identified. GO analysis classified these targets into cellular components, molecular functions, and biological processes, with “signal transduction”, “signaling” and “ADP binding” as the top biological processes (Figure 5a). Based on the expression profiles across different tissues, Fuzzy C-Means (FCM) clustering analysis grouped the target genes into four clusters: clusters 1 and 4 contained genes preferentially expressed in leaves and those not expressed in leaves, respectively; cluster 2 comprised the genes highly expressed in cambia; and cluster 3 included the genes preferentially expressed in reproductive tissues. GO analysis revealed distinct enriched terms for each cluster, indicating the expression and function differentiation of the target genes (Figure 5b,c).

Conserved miRNA-target pairs, such as miR159-*GAMYB*, miR160-*ARF*, miR156-*SPL*, and miR397-*LAC* were identified, suggesting the functional conservation of these regulatory mechanisms. A significant number of genes involved in reproductive development (e.g., *EMS1*, *SPL10*, *GAMYB*) [34,35,36,37,38] and disease resistance (e.g., *ROQ1*, *RUN1*) [39,40] were identified as miRNA targets in *G. biloba* (Table S6). The miRNA families targeting reproductive development-related genes include conserved families such as miR156, miR159, miR160, miR396, and miR529. miR482 and miR395 family members targeted 39 and 5 *ROQ1*, respectively, potentially regulating disease resistance. Notably, 12 miRNAs, including three novel ones, were found to target the biosynthetic genes of flavonoids and terpene trilactones (TTLs), which are the two major bioactive secondary metabolites in *G. biloba* (Figure 5d). Among these genes, *Levopimaradiene synthase* (*LPS*), a key gene family for TTL biosynthesis, was targeted by the highest number of miRNAs. Five miRNAs targeted four flavonoid biosynthesis genes. These findings highlight the critical regulatory role of miRNAs in secondary metabolism in *G. biloba*.

### 3.5. Multiple Regulatory Networks of miRNAs and phasiRNAs in G. biloba

**Figure 6:**
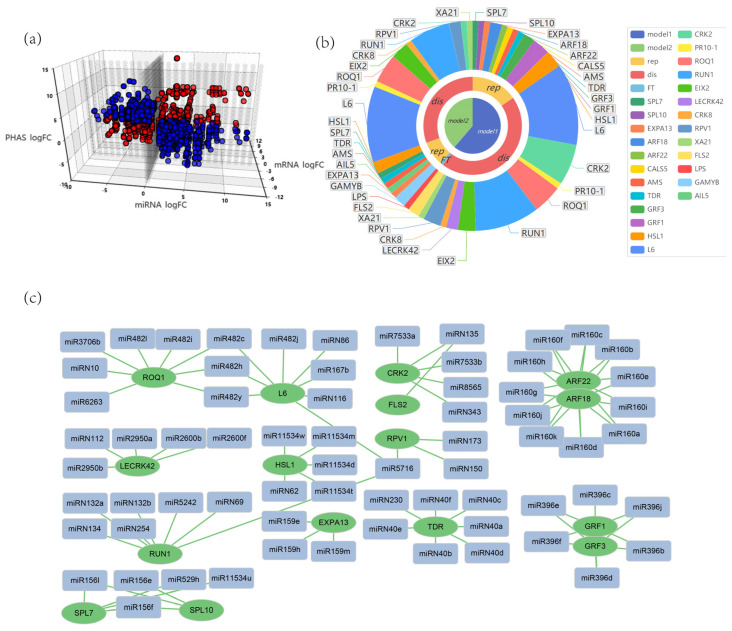
Regulatory network of miRNAs-mRNA-PHAS in *G. biloba*. (**a**) Expression profiles showing the fold changes (FC) of miRNAs, their corresponding target genes, and the triggered PHAS loci. Each point represents a potential interaction pair. Blue dots indicate miRNA regulation without phasiRNA involvement (Model 1), while red dots represent miRNA and phasiRNA co-regulation (Model 2). (**b**) Regulatory networks involved in reproductive development (rep), disease resistance (dis), and the biosynthesis of flavonoids and terpene trilactones (FT) in both models. (**c**) The regulatory network of Model 1 in *G. biloba* involves only miRNAs and excludes phasiRNAs. Blue rectangles represent miRNAs, while green circles represent target genes.

Combining transcriptome data from the same sample with sRNA sequencing, we analyzed miRNA-mRNA expression patterns. The samples were divided into reproductive tissues (ovules, embryos, male flowers, and female flowers) and vegetative tissues (leaves and cambia), from which negatively correlated miRNA-mRNA pairs were identified. By incorporating the triggering relationship with PHAS loci, a total of 5905 potential miRNA-mRNA-PHAS networks were identified, which formed two distinct models: one regulated solely by miRNAs (2352 pairs) (Figure 6a and Table S7) and another regulated by both miRNAs and phasiRNAs (3553 pairs) (Figure 6a and Table S8). Most miRNA-mRNA-PHAS modules lacked annotation, indicating a complex and largely unexplored regulatory network. However, the annotated genes revealed key functions: both modules predominantly target disease-resistance genes, suggesting that miRNAs and phasiRNAs may act synergistically to enhance the stability and precision of defense responses (Figure 6b). In contrast, the regulation of developmental genes differs between the two types. Genes like *GAMYB* and *HSL1* are regulated solely through the miRNA-mRNA-PHAS modue, while *GRF1/3*, *ARF18/22*, and *SPL7/10* are exclusively targeted by miRNAs (Figure 6c). This indicates layered regulatory strategies for balancing developmental processes and stress responses in *G. biloba*.

**Figure 7:**
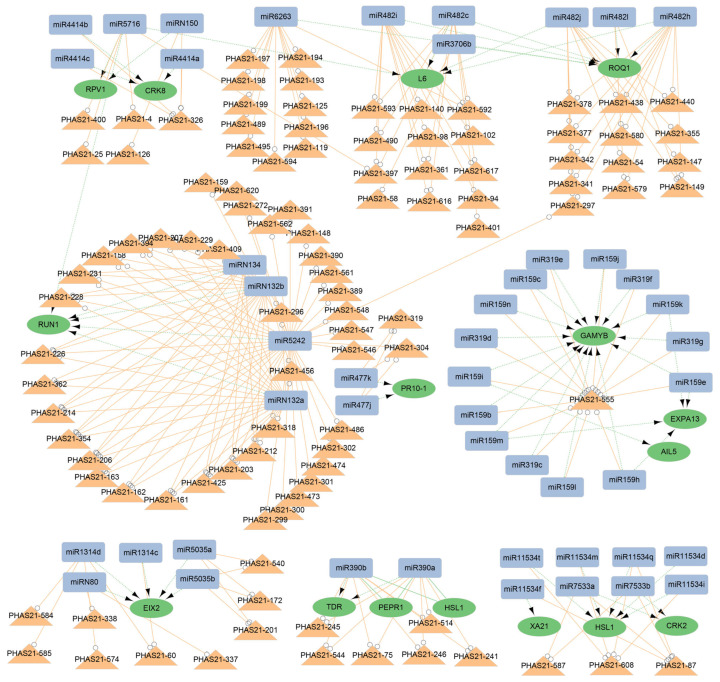
Regulatory network of miRNAs-mRNA-PHAS in Model 2 of *G. biloba*. Blue rectangles represent miRNAs, green circles represent targe genes, and orange triangles represent PHAS loci. The green dashed line indicates the negative correlation between miRNA and mRNA, whereas the orange solid line indicates the positive correlation between miRNA and phasiRNA.

In both models, disease-resistance genes exhibit complex, multilayered regulation, whereas genes associated with reproductive development are subject to more specific regulatory control. In the miRNA-only model (Model 1), developmental genes such as *GRF1/3*, *ARF18/22*, and *SPL7/10* are regulated by specific miRNA families—for instance, *GRF1/3* by miR396 and *ARF18/22* by miR160. In contrast, disease-resistance genes like *ROQ1*, *L6*, and *RPV1* are regulated by multiple miRNAs, forming intricate regulatory networks (Figure 6c). In the miRNA-phasiRNA co-regulatory model (Model 2), resistance genes like *ROQ1*, *L6*, and *RPV1* are also associated with various phasiRNA genes, suggesting a synergistic mechanism that enhances regulatory precision and robustness. Similarly, developmental genes like *GAMYB* exhibit dual regulation, underscoring the coordinated miRNA-phasiRNA control during the development of *G. biloba* (Figure 7).

### 3.6. Function of the miR159/miR319-PHAS Module in the Reproductive Development of G. biloba

**Figure 8:**
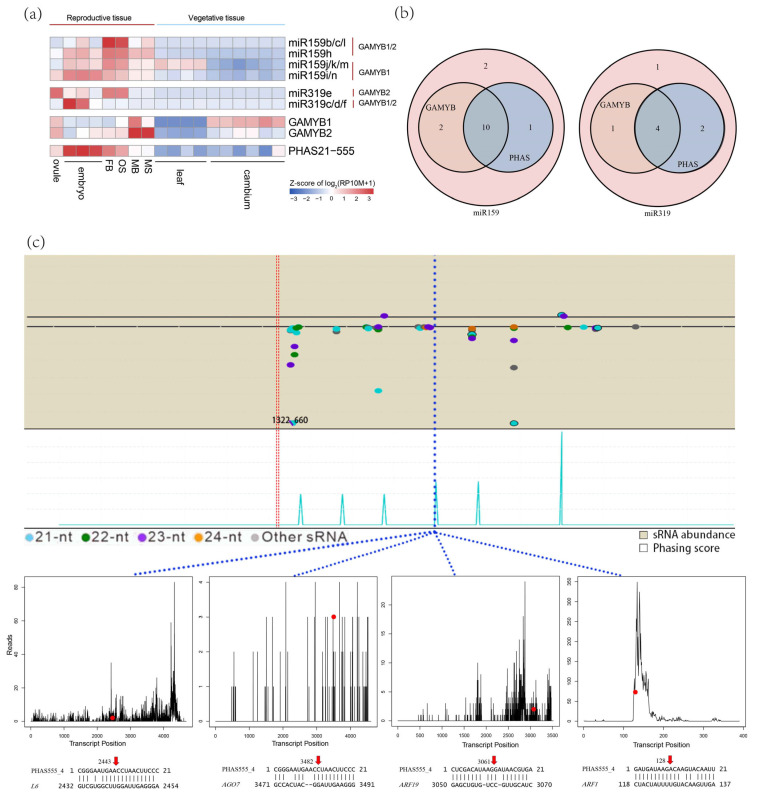
Reproductive regulation of miR159/miR319-PHAS module. (**a**) Tissue-specific expression of miR159/miR319, their target genes, and triggered phasiRNAs from PHAS loci. The color bar in the bottom right corner of the panel indicates the relative expression levels of phasiRNAs across different tissues. (**b**) Distribution of members in the miR159/miR319 family that target *GAMYB* and trigger the production of phasiRNAs from PHAS loci. (**c**) miR159/miR319 triggers the biogenesis of phasiRNAs from PHAS21-555, and the resulting four phasiRNAs regulated genes involved in reproductive development and stress resistance. The original data used for the plot is from the library of female flower buds (FB). Red dashed lines represent miR159/miR319 cleavage sites, while blue dashed lines represent cleavage sites of PHAS21-555-04/05/08. The 3′-5′ direction represents phasiRNAs, and the 5′-3′ direction represents mRNAs. The upper part represents the abundance of sRNA, and the lower part represents the Phasing score. The y-axis scale represents abundance. Each track’s y-axis scale appears in the upper left corner. Dots are colored by sRNA length, with red dashed lines showing miRNA cleavage sites. Triangle markers represent sRNAs exceeding the y-axis limits. The line plot below illustrates the validation of phasiRNA-mRNA interaction pairs using degradome data, with red dots marking the nucleotide positions cleaved in target genes. Red arrows represent mRNA cleavage sites, the x-axis represents the full length of the target mRNA, while the y-axis indicates the number of reads at specific locations.

The expression of the miR159/miR319 family in *G. biloba* showed a strong bias toward PHAS loci expression, with conserved roles in triggering PHAS555 to produce phasiRNAs. Among 15 miR159 and 8 miR319 members, 13 miRNAs targeted *GAMYB1* or *GAMYB2* (Table S9). Specifically, eight miR159 members were preferentially or exclusively expressed in female flowers (FB and OB) or/and ovule and embryos, but not male flowers (MB and MS). The same expression pattern was shown for miR319. Both miRNA families target *GAMYB* genes, which showed strong expression in male flowers (MB and MS) but not the female tissues (Figure 8a).

These miRNAs also trigger phasiRNA production from PHAS21-555, which, along with its triggers, shows preferential expression in embryos and female flowers (Figure 8a,b). To further investigate the potential biological functions of the miR159/miR319-PHAS21-555 module, the target genes of the three most abundant phasiRNAs (phasiR-555-04/05/08) were identified, including *L6* (evm.model.chr12.1424), *AGO7* (evm.model. chr10.1551), *ARF19* (evm.model.chr8.1790), and *ARF1* (evm.model.chr4. 1234) (Figure 8c). These genes are known to be involved in reproductive development or antiviral defense [41,42]. These findings suggest that the miR159/miR319-PHAS module may play a crucial role in reproductive development, potentially with sex-specific functions in *G. biloba*.

### 3.7. miR390-PHAS Module Involvement in Flavonoid Biosynthesis

**Figure 9:**
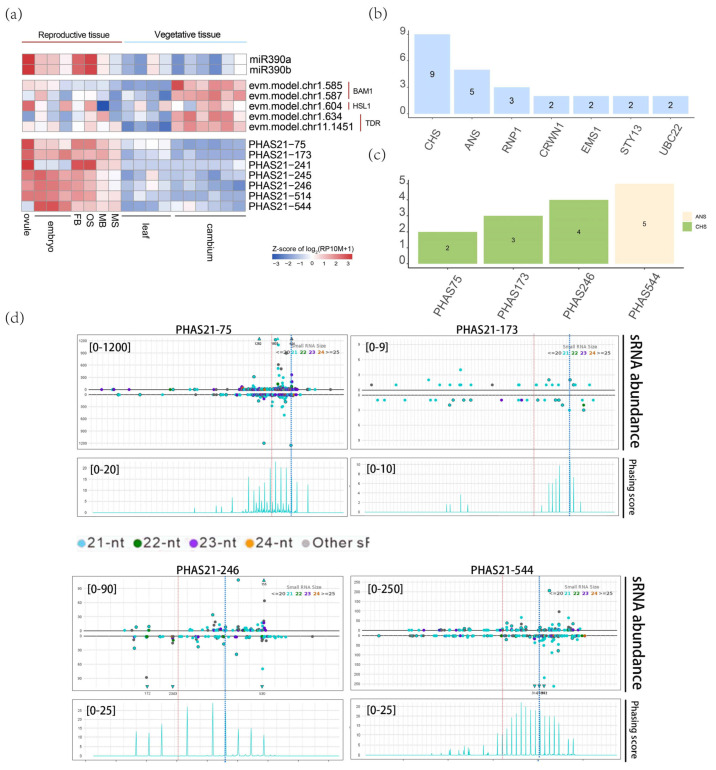
Flavonoid biosynthesis regulation by the miR390-PHAS module. (**a**) Expression of miR390a/b, their target gene expression, and the triggered phasiRNA from PHAS loci. The color bar in the bottom right corner indicates the relative expression levels of phasiRNA across different tissues. (**b**) The target genes of miR390a/b-triggered phasiRNAs, with the highest number of flavonoid biosynthesis genes *CHS* and *ANS*. (**c**) The number of *CHS* and *ANS* genes targeted by different phasiRNAs. (**d**) Four representative *CHS* and *ANS* genes cleavage by miR390a/b triggered phasiRNAs. The red dashed lines indicate the cleavage sites of miR390a/b at the PHAS loci, while the blue dashed lines represent the cleavage sites of phasiRNAs at the target genes. The upper part represents the abundance of sRNA, and the lower part represents the Phasing score. The y-axis scale represents abundance. Each track’s y-axis scale appears in the upper left corner. Dots are colored by sRNA length, with red dashed lines showing miRNA cleavage sites. Triangle markers represent sRNAs exceeding the y-axis limits.

Two miR390 were identified and both of which target *BAM1* (BARELY ANY MERISTEM 1, evm.model.chr1.585, evm.model.chr1.587), *HSL1* (HAESA-LIKE 1, evm.model. chr1.604), and *TDR* (TAPETUM DEGENERATION RETARDATION, evm.model.chr1. 634, evm.model.chr11.1451). miR390a/b accumulates exclusively in female flowers and ovules, while their target genes are expressed primarily in the cambium (Figure 9a). Previous studies have shown that *HSL1* and *BAM1* influence epidermal cell development by sensing different peptide signals, and *HSL1* may also be related to seed longevity [43,44]. On the other hand, *TDR* is a key component of the transcriptional cascade that regulates tapetum development and pollen wall formation in rice [45,46]. Therefore, miR390 may play an important role in cell development and secondary growth by regulating these target genes in *G. biloba*.

**Figure 10:**
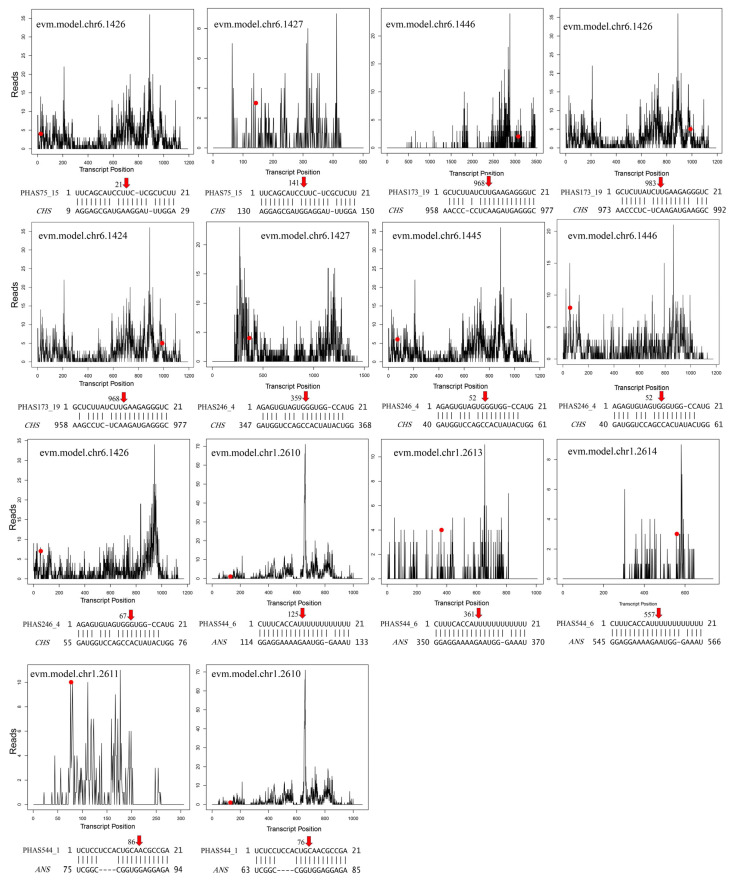
Degradation group-verified target genes of phasiRNA triggered by MiR390 regulate flavonoid biosynthesis. The line plot illustrates the validation of phasiRNA-mRNA interaction pairs using degradome data, with red dots marking the nucleotide positions cleaved in target genes. Red arrows represent mRNA cleavage sites, the x-axis represents the full length of the target mRNA, while the y-axis indicates the number of reads at specific locations.

Both miR390a and miR390b triggered the production of phasiRNAs from PHAS21-75, PHAS21-173, PHAS21-241, PHAS21-245, PHAS21-246, PHAS21-514, and PHAS21-544 (Table S8). Expression analysis showed that miR390 and trigger phasiRNAs have a similar pattern. By analyzing the downstream genes of the miR390-PHAS module, we found that phasiRNAs generated from the six PHAS loci were associated with nine *CHS* and five *ANS* genes (Figure 9b,c and Table S10), which encode two key enzymes for the flavonoid biosynthesis. Degradome data suggested potential the cleavage of these biosynthesis genes by phasiRNA (Figure 9d and Figure 10), which has not been reported, suggesting the new regulatory roles of the miR390-PHAS module and also the novel regulatory way of the secondary metabolites in *G. biloba*.

## 4. Discussion

### 4.1. miRNAs and miRNAs-phasiRNAs Regulatory Models in Ginkgo biloba

We compiled all 16 previously published studies on *G. biloba* sRNAs [13,15,16,17,18,19,20,21,47,48,49,50,51,52,53,54] and conducted a systematic analysis of the miRNAs and phasiRNAs in *G. biloba*. In this study, 342 known miRNAs were identified, with 249 overlapping with prior findings, confirming the reliability of our sequencing data. Additionally, we discovered 404 novel miRNAs in *G. biloba*. These newly identified miRNAs were predominantly from conserved miRNA families, including miR482 (13 members), miR169 (13 members), miR2600 (9 members), miR159 (8 members), miR477 (6 members), and miR395 (5 members). This study expands the small RNA database and provides new insights into the miRNA diversity of *G. biloba*. Our study reveals both conserved and divergent functional roles of miRNAs in *G. biloba* compared to other gymnosperms. While miR159 was reported to mediate ABA-responsive somatic embryogenesis in *Larix leptolepis* [8], we found it primarily regulates reproductive organ development via the *GAMYB* pathway in *G. biloba*. The conserved miR156-*SPL* regulatory module, observed here and in *Larix kaempferi* meristem aging [55,56], underscores its universal role in gymnosperm developmental timing. Since no reports of phasiRNAs in *G. biloba* have been published so far, we compiled a database of reported phasiRNAs in other plants. For example, 1204 phasiRNA loci were identified in *Arabidopsis thaliana* [57], and 2026 phasiRNA loci were identified in *Norway spruce*, though only 431 loci were suitable for further analysis [58]. In *grasses*, 1868 21-nt phasiRNA loci and 1723 24-nt phasiRNA loci were reported [59]. In rice (*Oryza sativa*), a total of 2338 21-nt phasiRNA loci and 172 24-nt phasiRNA loci were identified [60], Studies have also revealed the critical role of phasiRNAs in plant reproductive development. For instance, in maize, 463 21-nt phasiRNA loci and 176 24-nt phasiRNA loci were identified, with 24-nt phasiRNAs specifically accumulating in anthers and mature pollen [10], Similar results were observed in *wheat* and *barley*, where researchers identified 12,821 phasiRNA loci and 2897 phasiRNA loci, respectively. These findings were also validated in *G. biloba*, where 24-nt phasiRNAs were found to accumulate in reproductive tissues. Additionally, we identified 620 PHAS loci capable of producing 21-nt phasiRNAs and 33 loci capable of generating 24-nt phasiRNAs in *G. biloba*, broadening the scope of sRNA research in this species.

In our study, 746 miRNAs and 654 PHAS loci were identified in *G. biloba*, of which 526 PHAS loci (80%) were found to be triggered by 515 miRNAs (69%), implying that most of the miRNAs and phasiRNAs were co-regulatory. We refer to the regulatory model that involves only miRNAs, without phasiRNAs, as model 1 (Figure 6c). In this model, growth-related genes demonstrated distinct and specific regulation, leading to precise expression patterns. The *ARF* (Auxin Response Factor) and *GRF* (Growth-Regulating Factor) gene families are pivotal in plant growth and development [61,62]. *ARF* genes regulate auxin signaling, which is essential for organ morphogenesis and cell differentiation. Specifically, miR160 targets certain *ARF* family members to control root and leaf development, ensuring morphological stability and adaptability [63]. On the other hand, *GRF* genes primarily control cell proliferation and expansion, playing a significant role in leaf and flower development. miR396 regulates the rate of cell division and expansion by repressing *GRF* gene expression, thereby influencing organ morphology [64]. Both *ARF* and *GRF* gene families exhibit a high degree of specificity and precision in their regulatory mechanisms. Our research indicates that *ARF22* and *ARF18* are predominantly regulated by the miR160 family, while *GRF1* and *GRF3* are controlled by the miR396 family. These miRNA families achieve precise control of gene expression by specifically targeting their respective genes during different developmental stages and in various tissues. Notably, although many genes are regulated by phasiRNAs during development, our findings suggest that the expression of *ARF* and *GRF* genes is exclusively governed by miRNAs, with no involvement of phasiRNAs (Figure 6c). This highlights a highly specific role of miRNAs in regulating these gene families, implying that phasiRNAs may not play a significant or necessary role in controlling these growth-related genes. The regulatory functions of miR160 and miR396 in *ARF* and *GRF* gene expression reveal a streamlined mechanism of gene regulation in plants. This further emphasizes that the role of phasiRNAs in this particular pathway may be limited, underscoring the importance of miRNAs in maintaining precise control over key developmental processes.

In recent years, miRNAs have garnered significant attention for their pivotal role as key regulators of plant stress resistance. By modulating the expression of target genes, miRNAs enable plants to effectively respond to a wide array of biotic and abiotic stresses. During viral infections, plants employ miRNA-mediated gene silencing mechanisms to suppress viral replication and spread. For instance, studies have shown that the miR482 and miR2118 families are involved in regulating antiviral RNA silencing complexes [65]. Additionally, miR393 regulates F-box protein genes during pathogen infections, thereby inhibiting pathogen-associated hormone signaling pathways and bolstering immune responses [66]. Moreover, various miRNA families, including miR160 and miR398, regulate resistance genes to prevent excessive immune responses during viral infections, thus maintaining a balance between defense and growth [67]. miRNAs also play essential roles in plant responses to abiotic stresses, such as drought and salt stress. Research has revealed that miR159 is crucial in drought resistance, as it targets MYB transcription factors to modulate downstream stress-related genes [35,68]. miR828/858-MYB may play a key role in regulating needle discoloration. Under cold conditions, they negatively regulate flavonoid biosynthesis [69]. PhasiRNAs also contribute significantly to plant pathogen defense. Studies indicate that phasiRNAs target NBS-LRR resistance genes to fine-tune immune responses, thereby enhancing disease resistance [70]. In crops like tomato and rice, phasiRNAs regulate the expression of stress-responsive genes, providing protection during environmental stress [9]. Furthermore, phasiRNAs can form complexes with AGO proteins, leading to the silencing of pathogen-responsive genes [67].

Our findings indicate the possible existence of a unique and complex regulatory network associated with stress-resistance genes in *G. biloba*, which includes key players such as *RUN1*, *RQQ1*, *L6*, *RPV1*, and *CRK8*. These genes are subject to dual regulation by both miRNAs and phasiRNAs, exhibiting a high degree of regulatory complexity. Multiple families of miRNAs and phasiRNAs collaboratively regulate these genes, suggesting a sophisticated and interwoven network. This dual regulatory mechanism may provide the plant with a flexible and dynamic system for modulating gene expression. While our findings point to the potential role of this network in stress responses, it is important to note that our analysis was conducted under normal growth conditions. The direct involvement of these miRNA-phasiRNA pathways in biotic and abiotic stress responses remains to be validated. Nevertheless, the complexity of the network we observed implies that *G. biloba* may possess a highly adaptable regulatory framework, which could modulate gene expression levels through multi-layered, multi-pathway feedback mechanisms under stress conditions. This coordinated regulatory system may enhance the plant’s resilience, allowing it to effectively respond to environmental challenges. Further studies involving stress treatments are needed to confirm these regulatory roles during biotic and abiotic stress responses.

### 4.2. Typical miRNA-PHAS Modules in G. biloba

PhasiRNAs were initially discovered in *Arabidopsis thaliana* as small RNAs originating from specific genomic regions. These regions are typically associated with repetitive sequences related to protein-coding genes and were originally categorized as “small interfering RNAs” [6,71,72]. PhasiRNAs regulate plant gene expression by cleaving target mRNAs, playing a critical role in controlling growth, development, stress responses, and floral organ formation [73]. In maize, phasiRNAs have been implicated in lead stress tolerance [74]; in rice, they are closely associated with disease resistance and reproductive organ development [75]; and in tomatoes, phasiRNAs enhance disease resistance by targeting genes involved in pathogen responses [65]. The biogenesis of phasiRNAs necessitates key genes such as *DCL4* and *RDR6*, which process long double-stranded RNA into 21 or 24-nt phasiRNAs [76]. The production of these small RNAs typically requires prior miRNA-mediated mRNA cleavage [73]. This miRNA-triggered phasiRNA biogenesis pathway enables miRNAs and phasiRNAs to collaboratively regulate plant development and environmental responses [77]. For example, in *longan*, the miR482-PHAS module is predicted to be involved in squalene synthase production [11]; in *rice*, the miR2118-PHAS module cooperates with ARGONAUTE (AGO) proteins to regulate thermosensitive male sterility [78]; and in *legumes*, the miR1514-PHAS module has been confirmed to participate in post-transcriptional regulation [79]. By comprehensively analyzing sRNA libraries from various tissues of *G. biloba*, we have constructed a detailed sRNA regulatory network and identified two typical miRNA-PHAS modules.

The first module involves the miR159/miR319-PHAS network. MicroRNA159 (miR159) targets regulatory genes known as *GAMYB* or *GAMYB*-like genes through highly conserved miR159 binding sites. *GAMYB* genes, which encode the *MYB* domain transcription factor, are key regulators of plant reproductive development, playing a crucial role in pollen formation and the development of male reproductive organs [80]. These transcription factors transduce gibberellin (GA) signals in the seed aleurone layer and anther tapetum, influencing reproductive growth in plants [81]. On the other hand, miR319 is well-known for regulating the TCP family of transcription factors [82], thereby participating in leaf morphogenesis [83] and responses to environmental stresses such as cold, salt, and drought [84]. Previous studies have highlighted functional similarities between miR159 and miR319, For instance, Hu et al. [85] found that both miRNAs contribute to late-stage anther development and promote pollen abortion. However, Palatnik et al. [86] reported spatial and temporal expression patterns and significant differences in their binding abilities and regulatory efficiency with respective target genes. Notably, miR159 and miR319 are not only functionally similar but also closely related in terms of evolution. They are unique and closely related miRNA families in plants, sharing a common ancestor and being widely conserved across plant species [87]. Under certain conditions, they exhibit cross-targeting effects [88]. For instance, in *Brassica campestris*, the overexpression of MIR159a and MIR319c not only participates in late anther development but also promotes pollen abortion, with MIR319c partially compensating for the functional loss of MIR319a during flower development [85]. In rice (*Oryza sativa* L.), miR159 and miR319 jointly regulate growth and stress responses, highlighting their critical roles in plant development and environmental adaptation. In *G. biloba*, we identified 23 miR159 and miR319 loci, most of which target *GAMYB* genes and collectively trigger the production of phasiRNAs from a common PHAS locus. Additionally, degradome sequencing revealed the post-transcriptional regulatory mechanism of the miR159/miR319-PHAS21-555 module. Our findings indicate that these phasiRNAs are associated with genes involved in antiviral responses or reproductive development. Overall, our results suggest that the multilayered regulatory network involving these miRNAs and phasiRNAs, as well as their interactions with target genes, plays a crucial role in reproductive development in *G. biloba*. This synergistic miRNA-phasiRNA regulatory mechanism reflects the plant’s remarkable flexibility and precision in gene expression during sex differentiation, enabling rapid responses to developmental demands and environmental changes.

The second module is the miR390-PHAS network, which is possibly involved in the regulation of flavonoid biosynthesis. miR390 is widely recognized for triggering the biogenesis of TAS3-derived tasiRNAs in *Arabidopsis* [6,71,72]. It has also been reported to participate in lateral root development [89] and assist crops in resisting aluminum toxicity in acidic soils [90], as well as play a role in reproductive organ development [91]. Flavonoids, unique secondary metabolites in *G. biloba*, have attracted significant attention due to their medicinal value and role in stress resistance [92]. Studies show that the biosynthetic pathway of flavonoids directly influences the accumulation of its pharmacologically active compounds [93]. Additionally, flavonoids play an important role in *G. biloba*’s stress resistance, helping the plant defend against various biotic and abiotic stresses such as drought, pests, and UV radiation [94]. However, our research reveals that miR390 does not directly target genes involved in flavonoid biosynthesis. Instead, it indirectly participates in developmental regulation by targeting other development-related genes such as *HAESA-LIKE1* (*HSL1*), *BAM1*, and *TDR INTERACTING PROTEIN 3* (*TDR*), which are primarily associated with organ and flower development. These genes are predominantly expressed in the cambium, while the miRNA accumulates in reproductive tissues. Notably, although miR390 does not directly regulate flavonoid biosynthesis genes, the phasiRNAs triggered by miR390 play a critical role. Our findings indicate that miR390a/b triggers PHAS loci, which include nine phasiRNAs targeting *CHS* (chalcone synthase) and five targeting *ANS* (anthocyanidin synthase). These enzymes are key to flavonoid biosynthesis, with *CHS* acting as the initial enzyme in flavonoid biosynthesis, and *ANS* being involved in the modification and final synthesis of secondary metabolites [95]. In summary, this dual-layered regulatory mechanism—where miR390 regulates developmental genes and phasiRNAs precisely target metabolism-related genes—forms a complex network that enables *G. biloba* to coordinate its development and metabolism. This mechanism not only demonstrates the synergistic roles of miRNA and phasiRNA in *G. biloba* but also provides new insights into the relationship between secondary metabolism and developmental regulation.

## 5. Conclusions

The integrated analysis of miRNA profiling and degradome sequencing across various tissues of *Ginkgo biloba* reveals two distinct models of miRNA-mediated regulation. The first model involves regulatory pathways where only miRNAs are involved, without the participation of phasiRNAs. In this model, the regulation of auxin-related factors is particularly prominent. The second model is characterized by the co-regulation of both miRNAs and phasiRNAs, primarily targeting stress-responsive genes. This finding highlighted the complex and multi-layered regulatory mechanisms that plants employ to adapt to environmental challenges and developmental requirements. Furthermore, we identified two miRNA-PHAS modules associated with the growth and development of *G. biloba*. The first is the miR159/miR319-PHAS module, which plays a crucial role in reproductive development. The second is the miR390-PHAS module, which is potentially involved in the regulation of flavonoid biosynthesis. These modules highlight the sophisticated regulatory networks that govern key biological processes in *G. biloba*.

## Data Availability

The degradome data accession ID is PRJCA038234 (National Genomics Data Center, Beijing, China). The sRNA sequencing data generated as part of this study are deposited in the NCBI Sequence Read Archive (SRA), accession BioProject number PRJNA903548.

## Supplementary Materials

The following supporting information can be downloaded at https://www.mdpi.com/article/10.3390/plants14111650/s1, Figure S1: Expression heatmap of all identified known miRNAs; Figure S2: Expression heatmap of all identified Novel miRNAs; Figure S3: Expression heatmap of all identified 21-nt phasiRNA-producing loci; Figure S4: Expression heatmap of all identified 24-nt phasiRNA-producing loci; Table S1: sRNA-seq and RNA-seq data collections; Table S2: miRNAs in *G. biloba*; Table S3: PHAS loci in *G. biloba*; Table S4: miRNA-PHAS triggering relationship; Table S5: Reproductive tissue preferentially expressed miRNA-PHAS triggering relationship; Table S6: The target genes of miRNAs in *G. biloba*; Table S7: Model 1 regulatory networks involved miRNAs but not phasiRNAs in *G. biloba*; Table S8: Model 2 regulatory networks involved both miRNAs and phasiRNAs in *G. biloba*; Table S9: Degradome sequencing results for miRNA; Table S10: Degradome sequencing results for 21-nt phasiRNA; Table S11: Degradome sequencing results for 24-nt phasiRNA.
